# Mediastinal follicular dendritic cell sarcoma: a rare, potentially under-recognized, and often misdiagnosed disease

**DOI:** 10.1186/s13000-019-0779-3

**Published:** 2019-01-15

**Authors:** You-Li Wu, Feng Wu, Cheng-Ping Xu, Guo-Lei Chen, Yu Zhang, Wei Chen, Xiao-Chu Yan, Guang-Jie Duan

**Affiliations:** Institute of Pathology and Southwest Cancer Center, Southwest Hospital, Third Military Medical University (Army Medical University), Chongqing, 400038 China

**Keywords:** Follicular dendritic cell sarcoma, Mediastinum, Radiology, Pathological diagnosis, Prognosis

## Abstract

**Background:**

Mediastinal follicular dendritic cell sarcoma (FDCS) is extremely rare. Due to potential under-recognization of this disease, it happens to be misdiagnosed, especially on core needle biopsy. We report 3 cases of mediastinal FDCS and provide a literature review to improve better understanding of the tumor and to reduce misdiagnosis.

**Methods:**

Three cases of mediastinal FDCS in our clinic practice were studied, including their core needle biopsy and resected specimens, and those cases reported previously in English literature were retrieved and analyzed.

**Results:**

The core needle biopsy of case 1 showed a tumor reminiscent of classical Hodgkin’s lymphoma (CHL), while the resected mass was finally diagnosed with FDCS combined with hyaline-vascular Castleman’s disease. Both the biopsy and resected tissue of case 2 were constitutive of the clear epithelioid cells with marked atypia. In both cases, definitive diagnoses were not made on core needle biopsy. In case 3, there were some areas morphologically similar to CHL, and some areas contained ovoid to spindle-shaped tumor cells with fascicular pattern. The analysis of 43 cases of mediastinal FDCS showed the age of patients were from 16 to 76 years old, the male to female ratio was 1.5:1, the maximal tumor diameters were 3–17 cm. 18 cases were underwent preoperative biopsy, whereas 15 (83.3%) of which were misdiagnosed initially, often as lymphoma. 32 patients had available follow-up data, the rates of recurrence, metastasis, and mortality were 12.5, 18.8 and 28.1%, respectively. Current limited data suggested no statistical differences between adverse prognosis and gender, age, tumor size, necrosis, or different therapeutics, respectively.

**Conclusions:**

Mediastinal FDCS is a rare malignancy that has yet not been fully understood and been often misdiagnosed, particularly when making a diagnosis on core needle biopsy. Increased awareness of this enigmatic tumor is crucial to avoid diagnostic pitfalls.

## Background

Follicular dendritic cell sarcoma (FDCS) is a rare malignancy that originates from follicular dendritic cells featuring antigen-presenting activities [1]. Although the tumor has been found in both lymph nodes and extranodal sites, it is very scarce in the mediastinum, with approximately 40 cases so far reported in the English literature [2–32]. The clinical manifestations, radiological and pathological features of the tumor remain to be concluded. Due to potential under-recognization of mediastinal FDCS in clinicians and pathologists, it happens to be misdiagnosed or missed, especially on core needle biopsy, which may lead to a completely different treatment plan.

In this study, we presented detailedly 3 cases of mediastinal FDCS in our clinic practice, including core needle biopsy and resected specimens. Interestingly, our examination revealed that the morphology of core needle biopsy of 2 subjects were highly similar to those of classical Hodgkin’s lymphoma (CHL) and, therefore, had a high likelihood of misdiagnosis. Meanwhile, we reviewed 40 cases of mediastinal FDCS reported previously to explore their clinicopathological characteristics, and paid particular attention to the diagnostic difficulties and potential pitfalls in the preoperative biopsy. Furthermore, we analyzed, for the first time, the factors correlated to adverse prognosis of the tumor.

## Methods

### Clinical samples

We retrieved our pathological archives from 2006 to 2017 in 3 hospitals affiliated to the Third Military Medical University and Chongqing University Cancer Hospital, 3 cases of mediastinal FDCS were found. Case slides were reviewed again by 2 independent pathologists to confirm the diagnosis. Related clinical information was from the hospital medical records. Preoperative radiologic images were verified by radiologist in our hospital. Follow-up information was obtained by tracing the regular review of the patients.

### Histological examination

The 10% neutral buffered formalin-fixed, paraffin-embedded tissue blocks were cut into 4-μm-thick sections for hematoxylin and eosin (HE) staining and light microscopy.

### Immunohistochemistry

The immunohistochemical detection was performed on the automatic immunohistochemical staining device (BenchMark^XT^, Roche), and the antibodies were listed below in Table 1. The known positive sections were used for positive controls, and the phosphate buffer saline (PBS) buffer solution was used for negative control.

**Table 1:** Antibodies used for immunohistochemical staining and results

Antibody	Clone	Source	Results/Case No.		
			Case 1	Case 2	Case 3
CD21	EP64	ZSGB	+	+	+
CD23	EP75	ZSGB	+ (Focal)	+ (Focal)	+
CD35	EP197	ZSGB	+	+	–
CXCL-13	None	ZSGB	+	+	–
D2–40	D2–40	ZSGB	+	–	+
CD3	EP41	ZSGB	–	–	–
CD20	EP7	ZSGB	–	–	–
CD30	EP154	ZSGB	+ (scattered)	–	–
CD15	MMA + BY87	ZSGB	–	/	–
S-100	4C4.9	ZSGB	–	–	–
PLAP	EP194	ZSGB	/	+ (Weak)	/
SALL4	6E3	ZSGB	/	–	/
CK	AE1/AE3	ZSGB	–	–	–
P63	UMAB4	ZSGB	–	–	–
SMA	1A4	ZSGB	+ (Focal)	/	–
Desmin	D33	ZSGB	–	/	–
ALK	OTI1H7	ZSGB	–	/	/
EBV-LMP-1	CS1–4	ZSGB	–	–	–
Ki-67	MIB1	ZSGB	10%	30%	15%

*PLAP*, placental alkaline phosphatase; *CK*, Cytokeratin; *SMA*, smooth muscle actin; *ALK*, Anaplastic lymphoma kinase; *EBV-LMP-1*, Epstein-Barr virus latent membrane protein-1; +, positive; −, negative; /, None; *ZSGB*, Zhong Shan Golden Bridge, Beijing

### EBV-encoded RNA (EBER) in situ hybridization (ISH)

ISH for EBER was performed in 3 cases using a detection kit (Zhong Shan Golden Bridge, Beijing, China). The brown signal on the nuclei of the tumor cell was defined as a positive reaction. The tissue of nasopharyngeal carcinoma was used for positive control, and the reactive hyperplasia of the lymph node was used for negative control.

### Literature review

Mediastinal FDCS cases reported in English literature (before December 31, 2017) were retrieved by using PubMed, the references of which were also studied, so as to ensure the completeness of cases obtained from the literature. The clinicopathological information of the patients, such as the age, gender, tumor size and necrosis, imaging alterations, diagnosis and treatment process, prognostic information etc., was also collected.

### Statistical analysis

The Software Packages for Social Science 19.0 for Windows (SPSS, Inc. Chicago, IL, USA) was used for statistical analyses. Overall survival and disease-free survival rates were analyzed by Kaplan-Meier method. The correlation of the clinicopathological parameters with the adverse outcome (i.e. local recurrence, distant metastasis or death) were analyzed using the log-rank test. *P* value less than 0.05 was considered statistical significance.

## Results

### General information

The information of 3 patients with mediastinal FDCS in ours is summarized in Table 2. Cough and expectoration were the main manifestations of case 1 and 2, whereas chest tightness and chest pain were presented in case 3. No fever, fatigue, loss of appetite, or weight loss was observed in all cases.

**Table 2:** Summaries of 43 cases of mediastinal follicular dendritic cell sarcomas

Case No.	Age/sex	Tumour size (max)	Necrosis	Initial diagnosis	Treatment	Recurrence/Metastasis (mo)	Status	Follow-up (mo)
1 [3]	62/M	NA	Widespread	FDCS	Surg+ChT	Metastasis (24mo)	STD	24
2 [3]	46/M	NA	Widespread	FDCS	Surg+RT	No	NED	12
3 [3]	31/M	NA	No	FDCS	Surg	No	NED	10
4 [4]	44/F	3 cm	No	FDCS	ChT	NA	NA	NA
5 [5]	43/M	4 cm	No	Peripheral nerve sheath tumor	Surg	recurrences (36mo)	AWD	36
6 [6]	42/M	NA	NA	FDCS	Surg	No	NED	NA
7 [7]	35/M	4 cm	Yes	FDCS	RT + ChT	No	STD	7
8 [8]	76/M	10 cm	Yes	FDCS	Surg	No	NED	24
9 [9]	45/M	15 cm	No	Diffuse large B-cell lymphoma/Tuberculosis	Surg+ChT	multiple recurrences (156mo)	AWD	204
10 [10]	46/F	8.4 cm	No	Diffuse large B-cell lymphoma	RT + ChT	Metastasis (12mo)	AWD	24
11 [11]	43/M	13 cm	NA	FDCS	Surg	NA	NA	NA
12 [12]	45/F	8.4 cm	NA	Diffuse large B-cell lymphoma	ChT	No	AWD	22
13 [12]	41/F	5 cm	NA	FDCS	Surg+RT + ChT	No	NED	107
14 [13]	60/M	6 cm	NA	FDCS	Surg+ChT	No	STD	6
15 [14]	49/F	9 cm	NA	FDCS	Surg	No	NED	28
16 [15]	39/M	8 cm	NA	FDCS	Surg	NA	NA	NA
17[16]	23/M	8 cm	Focal	Malignant nerve sheath tumor	Surg+RT + ChT	Metastasis (45mo)	AWD	45
18 [17]	72/F	NA	NA	FDCS	Surg	No	NED	8
19 [18]	47/M	7.5 cm	NA	FDCS	RT	No	AWD	14
20 [19]	52/F	NA	Yes	FDCS	Surg	NA	NA	NA
21 [20]	42/M	6 cm	NA	Malignancy	Surg+RT	No	NED	20
22 [21]	68/M	4.5 cm	No	FDCS	Surg	No	NED	24
23 [22]	63/M	13.4 cm	Widespread	Malignant neoplasm(thymic epithelial origin)	Surg	NA	NA	NA
24 [23]	37/M	11 cm	Yes	necrotic material with no well-defined granulomas	Surg+RT + ChT	No	NED	6
25 [24]	62/M	7.5 cm	NA	No atypical cells were obtained	Surg+RT	No	STD	7
26 [25]	34/M	NA	NA	Low-grade non-Hodgkin lymphoma	Surg+RT + ChT	multiple recurrences (131mo)	AWD	312
27 [26]	39/M	6 cm	NA	FDCS	Surg+RT	No	NED	96
28 [27]	72/F	4.3 cm	NA	FDCS	RT	Metastasis (12mo)	STD	12
29 [27]	51/F	9.1 cm	NA	FDCS	Surg+RT	Metastasis (10mo)	STD	10
30 [27]	53/F	10.3 cm	NA	FDCS	Surg	No	NED	18
31 [28]	29/M	10 cm	No	NA	Surg+ChT	multiple recurrences (48mo)	AWD	102
32 [28]	25/M	13.5 cm	Widespread	NA	Surg+RT + ChT	No	NED	13
33 [28]	20/F	11.5 cm	No	NA	Surg	NA	NA	NA
34 [28]	61/M	14 cm	No	NA	Surg+RT + ChT	No	STD	57
35 [28]	40/F	4.2 cm	No	NA	Surg	NA	NA	NA
36 [28]	23/M	6.3 cm	Focal	NA	Surg	No	NED	NA
37 [29]	16/F	8 cm	No	FDCS	Surg	No	NED	24
38 [30]	46/F	11 cm	NA	Mediastinitis/Hodgkin lymphoma	Surg+RT + ChT	Metastasis (25mo)	STD	28
39 [31]	27/F	17 cm	NA	Suspicion of malignancy	ChT	No	STD	6
40 [32]	71/F	5.9 cm	NA	NA	Surg+RT	No	NED	15
41	52/F	5 cm	Focal	Suspicion of Hodgkin lymphoma	Surg+ChT	No	NED	60
42	46/M	13 cm	Widespread	Malignant neoplasm	Surg	NA	NA	NA
43	65/M	9.5 cm	No	FDCS	NA	NA	NA	NA

41–43 Present cases; *NA*, not available; *FDCS,* follicular dendritic cell sarcoma; Surg, surgery; *RT,* radiotherapy; *ChT*, chemotherapy; *NED*, no evidence of disease; *AWD,* alive with disease; *STD*, succumbed to disease

### Radiology

**Fig. 1:**
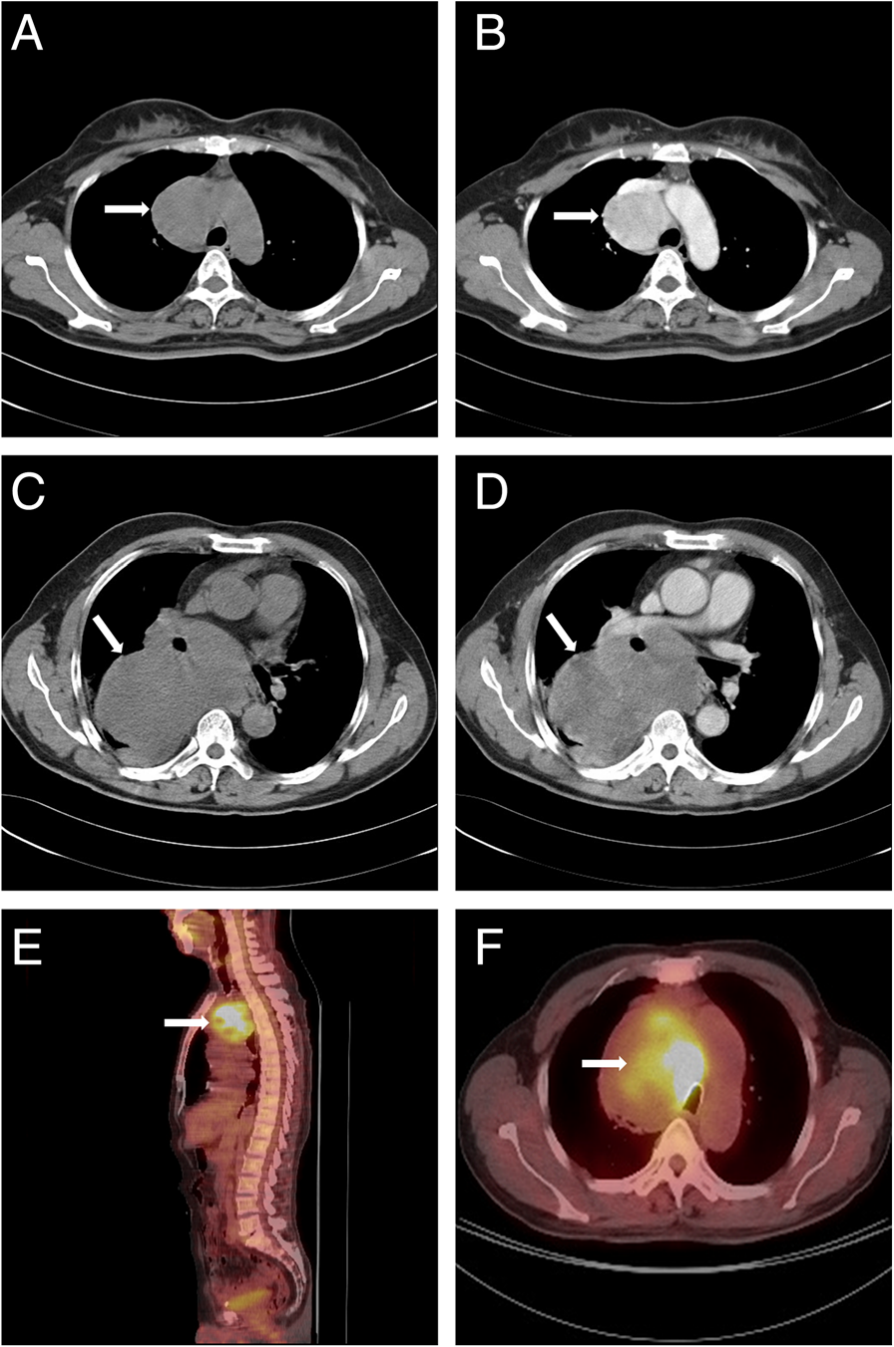
Axial CT of case 1 shows a mass with distinct border in the right upper anterior mediastinum (**a**), which presents an obviously heterogeneous enrichment after enhancement (**b**). Axial plain (**c**) and enhanced (**d**) CT of case 2 reveal that in the right posterior mediastinum, there is a giant lobulated shadow with moderately heterogeneous enhancement. Sagittal (**e**) and axial (**f**) positron emission tomography and CT of case 3 indicate a mass in the middle mediastinum with avid fluorodeoxyglucose uptake (SUVmax = 11.95)

Computed tomography (CT) images of case 1 showed that in the right upper anterior mediastinum adjacent to trachea, there was a mass of 5.0 × 4.9 × 4.7 cm, which had relatively distinct border and adhered tightly to the mediastinal vessels. After enhanced scan, the lesion had obviously heterogeneous enhancement (Fig. 1a, b). CT images of case 2 revealed that in the right posterior mediastinum, there was a giant lobulated shadow measured 13.0 × 12.6 × 7.6 cm. Post-contrast CT scaning displayed moderately heterogeneous enhancement of the lesion. The boundaries between the lesion and right pulmonary artery, esophagus and right bronchus were not clear (Fig. 1c, d). CT images of case 3 showed a mass in the middle mediastinum, 9.5 × 9.2 × 8.8 cm in size, which had clear boundary. The adjacent tracheae was slightly stenotic due to tumor compression. Positron emission tomography and CT (PET-CT) revealed significantly increased fluorodeoxyglucose (^18^F-FDG) uptake (SUVmax = 11.95), with most being internal uptake (Fig. 1e, f).

### Morphology and immunophenotype of the tumors

**Fig. 2:**
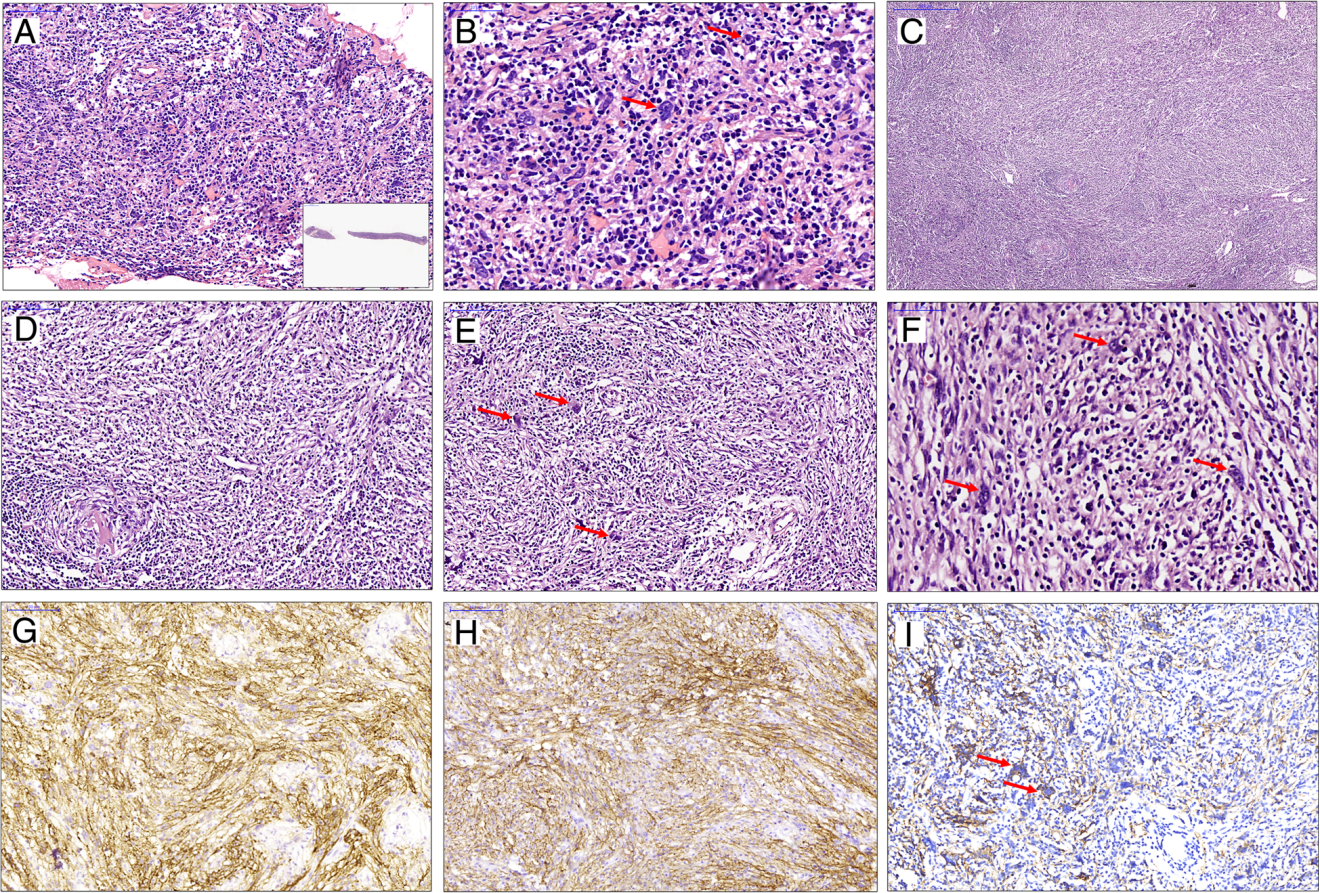
Case 1: HE staining of core needle biopsy specimen shows there are a few large round or ovoid cells scattered in the inflammatory background (**a**, inset: low-power view of needle biopsy), which are reminiscent of Hodgkin and Reed-Sternberg cells (**b**, arrows). Microscopic examination of resected specimens exhibit the classical morphology of hyaline-vascular Castleman’s disease (**c**, lower left area) and hyperplastic ovoid to spindle cells (**c**, upper right area). High-power view of figure C shows the transitional zones (**d**). Multinucleated giant cells and Hodgkin and Reed-Sternberg-like cells are disseminated in the lesions (**e**, **f**, arrows). Immunohistochemical staining shows the spindle cells (including some scattered large cells) are positive for CD21 (**g**), CD35 (**h**) and D2–40 (**i**, arrows)

Microscopic examination of the core needle biopsy of case 1 showed a few large round or ovoid cells scattered in the inflammatory background composed of lymphocytes, plasma cells and histocytes. These cells had delicate chromatin and large nuclei with distinct nucleoli, and were reminiscent of Hodgkin and Reed-Sternberg (HRS) cells (Fig. 2a, b, arrows). Immunohistochemical staining showed that these large cells were negative for cytokeratin (CK), P63, CD3, CD20, but a few were CD30 positive. Thus, the tumor was suspected to be classical Hodgkin lymphoma (CHL). However, the patient suffered haemorrhage during the puncture, which prompted emergency resection of the mediastinal mass. The resulting specimen was a grey mass with clear boundary and partial capsule. Microscopic examination showed the following findings: the main element of the tumor was composed of ovoid and spindle-shaped cells with fascicular, woven or storiform patterns, in the background, there were infiltrations of varying levels of lymphocytes, plasma cells, and eosinophils; some areas exhibited classical morphology of hyaline-vascular Castleman’s disease (HVCD). Between hyperplastic follicular dendritic cells in HVCD areas and surrounding spindle-shaped cells, there were transitional zones (Fig. 2c, d). Multinucleated giant cells and HRS-like cells were disseminated in HVCD areas, hyperplastic lesions of spindle-shaped cells, and the transitional zone in-between (Fig. 2e, f, arrows). Immunohistochemical staining showed that spindle-shaped cells (including some scattered large cells) expressed CD21 (Fig. 2g), CD23, CD35 (Fig. 2h), D2–40 (Fig. 2i, arrows), CXCL-13 and SMA at various degrees, a few large cells were CD30-positive, and the ki-67 labeling index was 10%. All of these cells were negative for CK, P63, Desmin, ALK, S-100 and EBV-LMP-1. Based on these results, the patient was diagnosed with FDCS combined with HVCD.

**Fig. 3:**
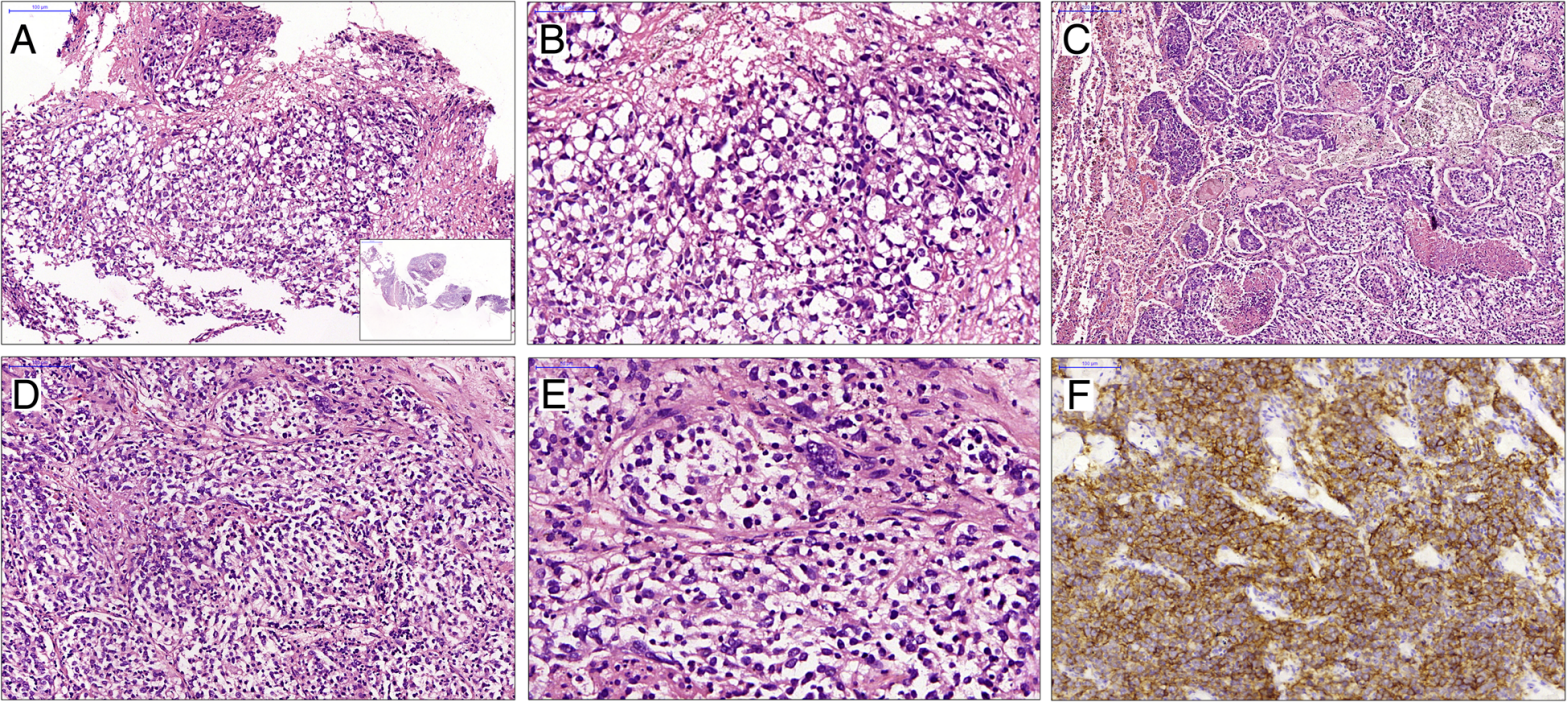
Case 2: HE staining of needle biopsy specimen reveals that round or ovoid tumor cells with clear cytoplasm are arranged into nests (**a**, inset: low-power view of needle biopsy), and high-power view shows the epithelioid cells with moderate to marked cytological atypia (**b**). Resected specimens reveal that epithelioid tumor cells with sheet or nest-like distribution invade into the pulmonary tissues (**c**, left area), and multifocal coagulative necroses are seen (**c**), moderate and high-power view of which show the clear epithelioid tumor cells (**d**, **e**) with CD21 expression (**f**), similarly to those of core needle biopsy

In case 2, microscopically, the needle biopsy specimens revealed that the tumor cells were round or ovoid with light red or clear cytoplasm, large and hyperchromatic nuclei, which were arranged into nests. Between these tumor nests, fibrosis and varying levels of lymphocytes infiltration were observed (Fig. 3a, b). Immunohistochemical staining showed that the tumor cells were negative for CK, P63, CD3, CD20, CD30, S-100, and weak positive for placental alkaline phosphatase (PLAP). So, a malignant tumor without further classification was made. During the surgery, the tumor was found to locate in the right posterior mediastinum and to have invaded into the right lung and oesophageal external wall. The resected specimen was a grey to white lobulated mass with clear boundary but no capsule. The tumor was tightly connected to partial pulmonary tissues; the cross-section was grey to brown with moderate texture, and contained bleeding and necrosis. Microscopic examination revealed that round to ovoid epithelioid tumor cells with marked atypia were tightly arranged into nested or a diffuse pattern. There were multifocal coagulative necrosis and a few dispersed lymphocytes in the background. The surrounding pulmonary tissue was invaded by tumor cells (Fig. 3c-e). Immunohistochemical analysis revealed that the tumor cells were positive for CD21 (Fig. 3f), CD35 and CXCL-13, focal positive for CD23, while negative for D2–40, CK, P63, CD30, SALL4, PLAP, EBV-LMP-1. The ki-67 labeling index was 30%. Thus the posterior mediastinal FDCS with right lung involvement was diagnosed.

**Fig. 4:**
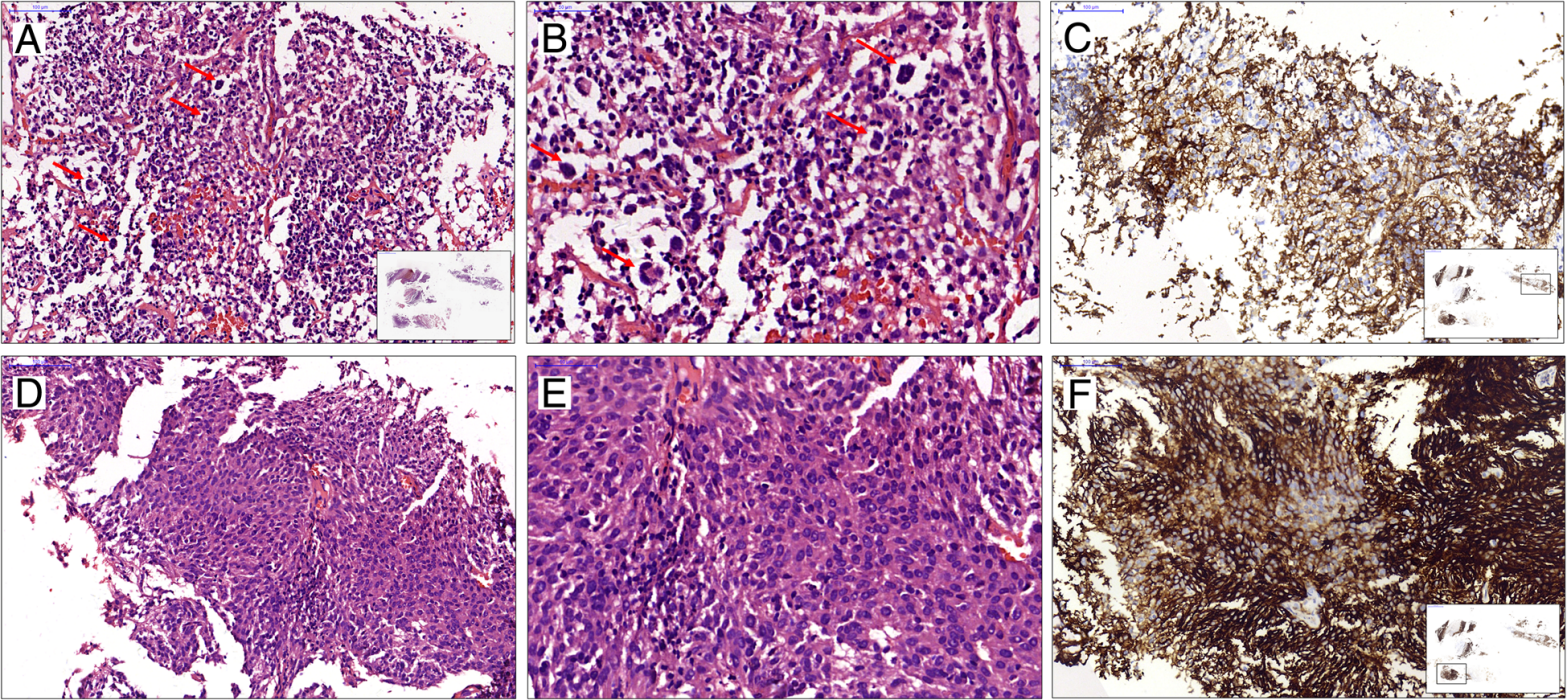
Case 3: HE staining of core needle biopsy specimen shows 2 different histopathological morphologies: one is that in the inflammatory background, there are scattered large round or ovoid cells resembling lacunar Reed-Sternberg cells (**a**, **b**, arrows, inset: low-power view of needle biopsy), another presents ovoid and spindle tumor cells with bundles and woven patterns (**d**, **e**), both of which are positive for CD23 (**c**, **f**, inset: low-power view of immunohistochemical staining of needle biopsy)

In core needle biopsy of case 3, two different histopathological morphology was identified, with some areas resembling that of case 1, specifically, in the inflammatory background composed of with lymphocytes, plasma cells and histocytes, there were scattered large round or ovoid cells resembling lacunar Reed-Sternberg (LHS) cells (Fig. 4a, b, arrows). In other areas, there were ovoid and spindle-shaped tumor cells with bundles and woven patterns, the tumor cells had abundant and slightly eosinophilic cytoplasm, and hyperchromatic nuclei (Fig. 4d, e). Immunohistochemical staining showed that tumor cells in both areas were positive for CD21, CD23 (Fig. 4c, f) and D2–40, while negative for CD35, CXCL-13, CK, P63, CD30, S-100, EBV-LMP-1. The ki-67 labeling index was 15%. The patient was diagnosed with FDCS.

### Results of ISH

The tumor cells were all negative for EBER by ISH in 3 cases.

### Literature review

Literature retrieved 40 cases of mediastinal FDCS (Table 2), plus with 3 cases in ours, generated a total of 43 cases for statistical analysis, including 26 males and 17 females. The ages arrange was from 16 to 76 years old (mean age 46 years, and median age 45 years). The maximal tumor diameters were 3–17 cm (mean 8.6 cm and median 8.2 cm). Of 24 cases, concurrent necrosis was found in 12 cases (including 5 cases with extensive necrosis), but another 19 subjects had no relevant records.

Among the 43 cases, 18 underwent preoperative needled biopsy, 15 of which (83.3%) were misdiagnosed initially. Mediastinal FDCS was often diagnosed as other types of tumor or non-tumor lesions, including lymphoma (7 cases), malignant tumor (without classification, 3 cases), malignant schwannoma (2 cases), undifferentiated carcinoma (2 cases), thymoma (1 case), germ cell tumors (1 case), or no atypical cells (2 cases).

**Fig. 5:**
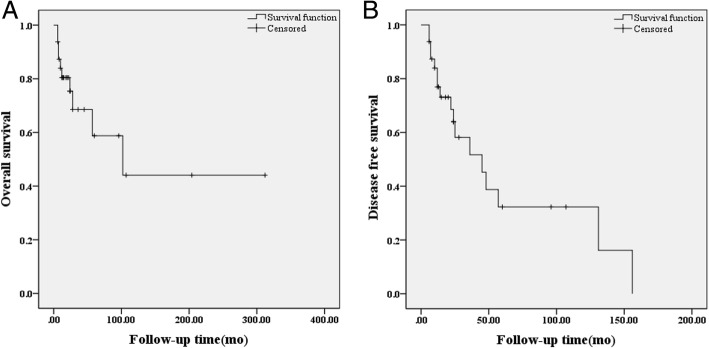
Survival curves of mediastinal FDCS, overall survival (**a**) and disease-free survival (**b**)

Available follow-up data was obtained from 32 subjects, the follow-up duration was 6–312 months, averaging 43 months. The rates of recurrence, metastasis, and mortality were 12.5, 18.8 and 28.1%, respectively. The 1-, 3-, and 5-year total survival rates were 80.4, 68.5, and 58.8%, respectively, and the 1-, 3-, and 5-year tumor-free total survival rates were 76.9, 51.7, and 32.3%, respectively (Fig. 5a, b).

### Prognosis associated factors

**Fig. 6:**
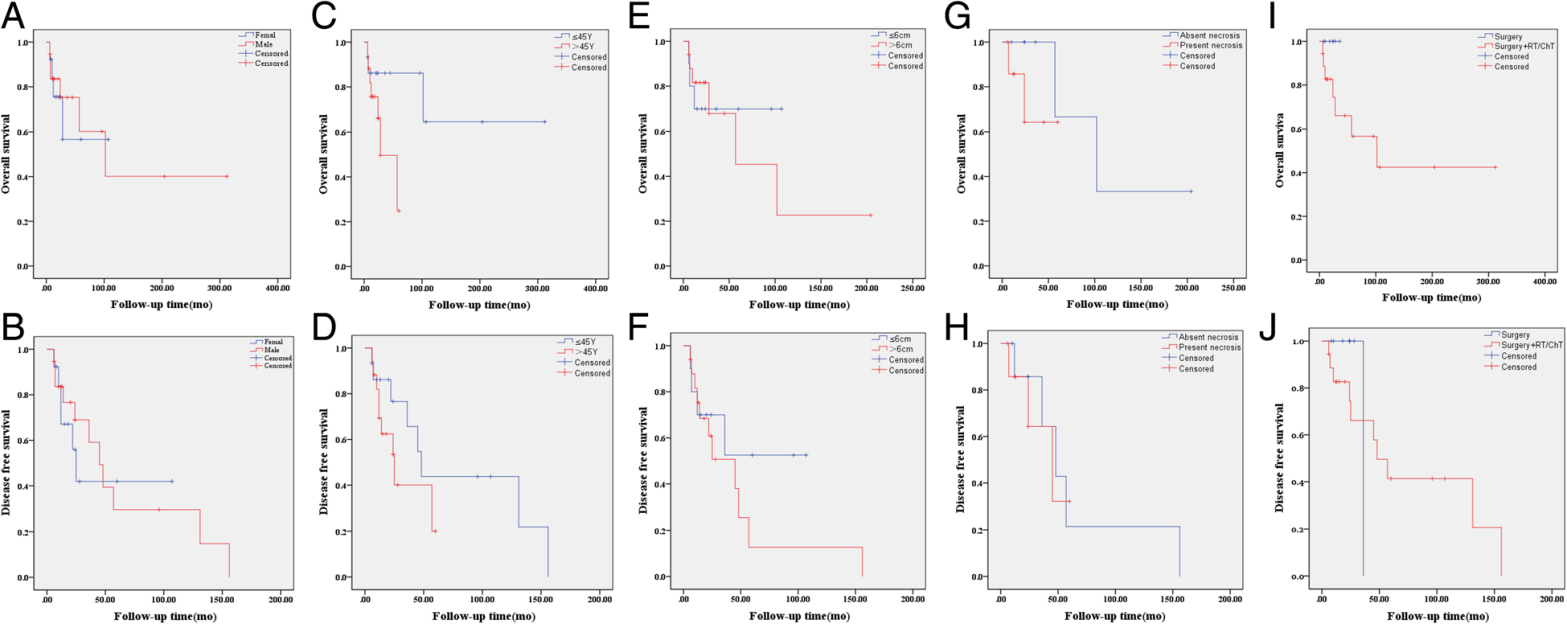
Survival curves of mediastinal FDCS. OS and DFS curves of FDCS with various gender (Male or Female, **a**, **b**), age (≤45y or > 45y, **c**, **d**), tumor sizes (≤6 cm or > 6 cm, **e**, **f**), necrosis situation (absent or present necrosis, **g**, **h**). OS and DFS curves of FDCS with different treatments (surgery alone or surgery followed by RT and/or ChT, **i**, **j**). OS, overall survival; DFS, disease-free survival. RT, radiotherapy; ChT, chemotherapy

Given the limitations in sample numbers and availability of clinical data (reliable data were not easy to obtain for mitotic figures and nuclear pleomorphism), we only analyzed the associations of poor prognosis with the following clinical factors: gender, age, maximal tumor diameter, and presence of necrosis. Our analysis revealed that there were no significant differences in either total survival rate or tumor-free survival rate within sex, age, tumor size and necrosis subgroups (Fig. 6a-h). Next, we compared prognosis differences between different treatments (8 underwent surgery only vs 18 underwent surgery plus adjuvant radiotherapy/chemotherapy), and there was no differences in either total survival rate or tumor-free survival rate (Fig. 6i-j).

## Discussion

Mediastinal FDCS is a rare disease. Currently, there is a lack of systematic, large-sample studies on its clinical manifestations, radio-pathological links, and prognosis-correlated factors. In this study, we analyzed a large group of mediastinal FDCS (43 cases), including patients admitted in our hospital and those from the literature [3–32]. The subjects were an average of 46 years old, and sex ratio was 1.5:1 (M:F). The clinical manifestations were not specific, but closely associated with the site of tumor occurrence. A few patients were occasionally found lesions during physical examination. Interestingly, 4 cases had paraneoplastic pemphigus (PNP) as the first clinical manifestation [9, 13, 24, 26], whereas 3 cases had myasthenia gravis (MG) as the main symptom [5, 15, 17]. Because MG is common in patients with thymoma, preoperative diagnosis is expected to be a challenge for these cases.

Among the 43 cases, 19 cases published with radiological image data, which indicated that the tumors often presented a round or lobulated shadow with clear border. After enhanced scan, the tumor showed obviously enhancement. The tumors were commonly relatively large in size. Calcification was found in 52.6% of the cases, although bleeding and necrosis were rare. It was proposed that if a mediastinal mass had clear border, calcification, but rare bleeding and necrosis, the condition was highly likely FDCS [11, 27]. Some cases may develop HVCD-like imaging alterations. For example, Case 1 in ours had concurrent HVCD and displayed obvious enhancement, which was highly similar to HVCD and practically indistinguishable from it in CT images [21]. Of note, Case 1 suffered haemorrhage during preoperative puncture, which also occurred for one of the cases in the literature [18]. Above-mentioned cases remind us if the tumor image displays apparent intensification after enhance scan, which indicates it may be rich blood supply and vulnerable to haemorrhage, the puncture risk assessment is necessary. There are very few reports on the PET-CT features of mediastinal FDCS (only 4 cases), with SUVmax values ranging from 2.7 to 11.4. The SUVmax of Case 3 was 11.95, indicating that the tumor had a relatively high metabolic level.

Most cases with mediastinal FDCS manifest the typical morphology of conventional FDCS. However, some cases may develop marked atypia or epithelioid or CHL-like features, which present a great challenge to diagnosis, particularly preoperative needle-based diagnosis. Of the 43 cases analyzed in this study, 18 received core needle biopsy but had an overall misdiagnosis rate of 83.3%, illustrating the extraordinary difficulty involved in identifying the disease. Core needle biopsy is of vital importance to mediastinal tumors, in particular for those that are difficult to remove surgically, because their subsequent therapeutics almost rely on the preoperative examination. Based on our diagnostic experience and literature, the causes of diagnostic difficulty may be attributed to the following: (1) mediastinal FDCS lacks unique clinical signs and may present unusual symptoms such as PNP or MG; (2) although radiologic image is indicative to a certain extent, its diagnostic value is limited; (3) the tumors are heterogeneous, often with concurrent inflammation and fibrosis, thus small needle specimen can not fully represent the whole tumor; and (4) mediastinal FDCS is so scarce that clinicians and pathologists often do not considered it during diagnosis. Hence, increased awareness of this rare mediastinal tumor and consideration of detection of the FDC-associated markers are crucial to avoid pitfalls.

An important scenario that should raise a red flag is that, based on the data from the literature and our experience, mediastinal FDCS is the most likely to be diagnosed as lymphoma (7/18 cases). Two of the biopsy specimens obtained in our hospitals also displayed a CHL-like appearance; specifically, such morphology refers to the scattered Reed-Sternberg-like large cells and multinucleated giant cells, which may express CD30, in a mixed inflammatory background. This critical issue can potentially be a diagnostic trap because once a patient is diagnosed as CHL, he/she likely receives unsuitable treatments. As for the differential diagnosis between the two entities, there are several points to note. (1) Reed-Sternberg-like large cells and CD30 positivity are very limited role, because these scattered large cells are often found in FDCS in different locations [2, 33, 34], and part of which could expression CD30 [35]. (2) It is the most important that when such lesion is presented in a needle biopsy of mediastinal mass, it should be considered not only the possibility of CHL but also that of FDCS. (3) The markers correlated to FDCS and CHL should be detected, including EBER ISH. If the large cells are positive for CD30, CD15, Mum-1, GATA3, PAX5 (weak positive) and EBER, but FDC-associated markers are negative, CHL is considered. On the contrary, if only CD30 and FDC-associated markers are positive, it should be considered as FDCS.

Previously, it was considered that adverse outcome of FDCS could be attributed to the following aspects: its intra-abdominal location, its large size (often > 6 cm in diameter), the presence of coagulative necrosis, the high proportion of mitotic figures (> 5/10 HPF), and the marked nuclear atypia. However, whether these factors are suitable for mediastinal FDCS is unclear. Current limited data suggested that none of the factors (gender, age, tumor size, and presence of necrosis) were correlated with the adverse outcome. Of course, an accumulated large sample size and long follow-up duration are needed to better investigate this issue.

Currently, there is a lack of consensus on treatment of FDCS, but radical resection is the predominant option, and adjuvant radiochemotherapy remains controversial [1, 25, 36]. In this study, a comparison of prognosis between the surgery only group and the surgery plus adjuvant therapy group found no differences in either total or tumor-free survival. However, it is note that the cases available for analysis are scarce, and the survival curves based on a compliation of small series and case reports, so the aboved results may be insufficiently valid. Thus, further studies are needed to better explore the effects of adjuvant therapy.

The aetiology of FDCS remains elusive. It has been reported that its occurrence is closely related to HVCD, which, rather than a reactive alteration in tumor stroma, is more likely a type of precancer lesion [28, 37]. Chan et al. reported a clinical case in which HVCD evolved into FDCS [38]. Of the 43 cases of mediastinal FDCS, 8 (18.6%) had concurrent HVCD. For example, case 1 had a transitional zone between the HVCD and the FDCS, corroborating that they might correspond to different phases of the same disease. Hence, when examining a patient with a putative mediastinal HVCD, it is important to maximize the sample areas and to perform a thorough microscopic examination, thereby avoiding potential FDCS lesions being missed. Epstein-Barr virus (EBV) infection is considered to be closely linked to the occurrence of inflammatory pseudotumor-like FDCS, but conventional FDCS seldom have concurrent EBV infection [33, 39]. Of the 43 cases, 10 were detected by EBER ISH, which were all negative.

Little is known about the pathogenesis of FDCS, only a few studies have been reported so far [35]. Go et al. performed BRAF sequencing and identified V600E mutations in 5 of 27 FDCS cases (18.5%) [40]. In our 3 cases, BRAF mutation analysis was also performed by fluorescent PCR, but no V600E mutation was detected *(data not shown)*. Recurrent loss-of-function alterations were revealed in tumor suppressor genes involved in the negative regulation of NF-κB (38%) and cell cycle (31%) through targeted sequencing [41]. Based on microRNA (miRNA) profiling of 31 FDCSs, Hartmann et al. identified two subgroups with high and low miRNA expression levels, which related to fibroblasts and myopericytomas or Castleman’s disease, respectively [42]. Two newly researches on the whole transcriptome have shed new light on the pathobiology of FDCS. Laginestra et al. demonstrated FDCS has a distinct FDC-related transcriptional profile which allows differentiation from other mesenchymal tumors. The results also provide evidence of a peculiar immune microenvironment associated with FDCS that may have clinical utility [43]. By whole transcriptome sequencing, Lorenzi et al. identified two novel FDC markers, the FDC secreted protein and serglycin, and proposed an efficient marker panel for the diagnosis of this enigmatic tumor [32].

## Conclusions

Mediastinal FDCS is very rare and lacks distinct clinical manifestations, which is a great challenge for diagnosis, especially on core needle biopsy. Mediastinal FDCS is the most likely to be diagnosed as lymphoma, which the differential diagnoses should be mostly focused on. Better understanding of this enigmatic tumor is important for augmenting its diagnostic accuracy.

## Abbreviations

ALK: Anaplastic lymphoma kinase
CHL: Classical Hodgkin’s lymphoma
CK: Cytokeratin
CT: Computed tomography
EBER: Epstein-Barr virus-encoded RNA
EBV: Epstein-Barr virus
EBV-LMP-1: Epstein-Barr virus latent membrane protein-1
EMA: Epithelial membrane antigen
FDCS: Follicular dendritic cell sarcoma
HVCD: Hyaline-vascular Castleman’s disease
IHC: Immunohistochemistry
ISH: In situ hybridization
MG: Myasthenia gravis
PET-CT: Positron emission tomography and CT
PLAP: Placental alkaline phosphatase
PNP: Paraneoplastic pemphigus
RNA: Ribonucleic acid
SMA: Smooth muscle actin
